# Three Types of Mammary Tumour Induced in Rats by Feeding with DMBA

**DOI:** 10.1038/bjc.1964.58

**Published:** 1964-09

**Authors:** P. M. Daniel, Marjorie M. L. Prichard

## Abstract

**Images:**


### THREE TYPES OF MAMMARY TUMOUR INDUCED IN RATS BY FEEDING WITH DMBA

P. M. DANIEL AND MARJORIE M. L. PRICHARD

*From the Department of Neuropathology, The Institute of Psychiatry, Maudsley Hospital, Denmark Hill, London, S.E. 5, and The Nuffield Institute for Medical Research, University of Oxford*

Received for publication May 1, 1964

IN 1961 Huggins and his colleagues (Huggins, Grand and Brillantes, 1961) reported that mammary tumours could be induced rapidly and reliably in rats by means of a single dose of 20 mg. 7,12-dimethylbenz(a)anthracene (English notation 9,10-dimethyl-1,2-benzanthracene) given by stomach tube. Since this method has such obvious practical advantages over that requiring repeated doses of 3-methylcholanthrene (Huggins, Briziarelli and Sutton, 1959 ; Dao and Sunderland,1959 ; Daniel and Prichard, 1961, 1964*c*) we decided to use it in continuing our studies on experimentally induced mammary tumours (Daniel and Prichard, 1963, 1964*a*). In this paper we report the results of our first group of experiments with this carcinogen.

### METHODS

Fifty-one female Sprague-Dawley rats, bred in our own laboratories, were used. After weaning the rats had no access to males at any time. At the age of 50 days ($\pm$ 1 day) they were given 20 mg. 9,10-dimethyl-l,2-benzanthracene (DMBA), dissolved, in 1 ml. sesame oil, by stomach tube. Thereafter the rats were palpated at weekly intervals to determine whether any tumours had developed. The presence of a palpable tumour was not recorded until a nodule as large as a pea (0·8–1·0 cm.) was felt. Soon after a tumour had attained this size a biopsy specimen was taken, under ether anaesthesia, for microscopic examination, and at this time 6 of the rats were subjected to hypophysectomy and 15 others to bilateral ovariectomy ; the remaining rats received no treatment ; in a few cases further biopsy specimens were taken of the tumours. The feeding and general care of the animals was as described in our previous papers. Most of the rats were kept for periods ranging from 6 to 15 months after the date of the carcinogen feeding, the termination of the experiment in each animal depending on various circumstances, e.g., the development of intercurrent infection, the excessive growth of a tumour or the need to search for and examine microscopically the remnant of a tumour which had regressed after one of the operations mentioned above. The rats were finally killed with chloroform, and with a few exceptions a detailed autopsy was carried out, all tumours that were found being taken for microscopic examination. In many instances tumours too small for identification by palpation were discovered to be present at autopsy. From most animals several macroscopically normal mammary glands were taken for histological study. A search for metastases was also regularly made.

The tumours and other tissues taken were fixed in formol-alcohol (10 per cent formalin, 60 per cent alcohol). Blocks were embedded in paraffin wax, and sections were cut at 7 $\mu$ and stained with haematoxylin and eosin and with haematoxylin and Van Gieson's mixture. Frozen sections were cut from some of the tumours and stained with Oil Red O.

### RESULTS

### *Incidence and nature of the tumours*

Of the 51 rats which had been given DMBA, 41 animals developed mammary tumours. In 38 of these animals the presence of one or more tumours was easily established by palpation, but many of the rats, particularly those kept for over 6 months, were found at autopsy to have additional mammary tumours which were usually too small to be felt through the skin (Fig. 8). In the other 3 rats the existence of tumour tissue was discovered only at autopsy. The remaining 10 rats of the series that had been given the carcinogen did not develop any palpable tumours during the period of their survival, which ranged from 6 to 52 weeks after the carcinogen feeding (the mammary glands of these animals were macroscopically normal but were not examined histologically to see whether small tumours were present).

The earliest time that the presence of a tumour was established by palpation was 8 weeks after the carcinogen had been given, and 7 rats had developed palpable tumours by the 11th week. However, in 25 animals palpable tumours did not appear until much later, the times being spread fairly evenly over the period from 6 to 12 months after the carcinogen feeding.

In all, 137 tumours were found to be present in the 41 rats. They were situated along the so-called milk-line at various levels from the neck to the lower abdomen. Microscopic examination of 135 of these neoplasms in 40 animals

---

### EXPLANATION OF PLATES

FIG. 1.—Non-secreting mammary adenoma, induced in a rat by DMBA, showing acini with many layers of cells in their walls. This type of tumour may also have the appearance seen in Fig. 2. H. and E. ×176.

FIG. 2.—Non-secreting mammary adenoma, induced by DMBA, showing a papillary pattern and a less dense appearance than that seen in Fig. 1. H. and E. ×176.

FIG. 3.—Milk-secreting mammary adenoma induced by DMBA in a virgin rat. The walls of the acini have only one layer of epithelial cells, and the lumen contains a substance resembling milk (see Fig. 6). Vacuoles are prominent in this substance and in the cytoplasm of the epithelial cells. This type of tumour is further illustrated in Fig. 5 and 7. H. and E. ×176.

FIG. 4.—Mammary fibro-adenoma induced by DMBA. H. and E. ×176.

FIG. 5.—Milk-secreting mammary adenoma in a virgin rat given DMBA. Note the general resemblance to Fig. 6. H. and E. ×176.

FIG. 6.—Mammary gland of a normal rat late in pregnancy, showing milk and vacuoles present in the lumen of the acini, as seen in the milk-secreting adenomata induced by DMBA (Fig. 3, 5 and 7). H. and E. ×176.

FIG. 7.—Mammary tumour of mixed type induced by DMBA. Milk-secreting adenomatous tissue is seen above, and fibro-adenomatous tissue below. H. and E. ×176.

FIG. 8.—A small mammary tumour (*T*) of milk-secreting type found to be present in a macroscopically normal mammary gland 48 weeks after DMBA. Some normal mammary tissue is seen below the tumour, and although the section does not show the nipple, the duct (*D*) leading to it is visible. H. and E. ×11.

Daniel and Prichard.

Daniel and Prichard.

showed that they were not all of the same type, but consisted of three main varieties. Twenty-three of the tumours, found in 19 rats, were ordinary adenomata, showing the characteristic histological features seen in adenomata induced by 3-methylcholanthrene (Daniel and Prichard, 1961, 1963, 1964*a*, *c*). Some of these tumours showed a very compact structure, the acini being crowded together in clumps and containing many layers of cells in their walls (Fig. 1), while others had a less dense appearance, the acinar walls being formed of fewer cells and the general pattern being of a papillary character (Fig. 2). These adenomata could usually be identified on palpation by their firm consistency.

Fifty other tumours, present in 18 rats, were also adenomata, but with a wholly different appearance. The walls of the acini were composed of a single layer of cells, and the lumen was filled with a basophilic substance resembling milk. Vacuoles were characteristically present in this substance and also in the cytoplasm of the epithelial cells (Fig. 3, 5 and 7); the contents of the vacuoles showed a positive reaction in sections stained for fat. The appearances resembled those seen in the mammary gland of a normal rat at the end of pregnancy (Fig. 6) or during lactation, except that in the tumours the epithelial cells were plumper, more interstitial tissue was present, and the arrangement of the acini was much more irregular than in the normal gland. On palpation through the skin it was usually difficult to distinguish these milk-secreting adenomata from the non-secreting adenomata described above, but when the former tumours were exposed milky patches were often seen on the surface beneath the capsule, and when cut across with a scalpel the tumours would exude a milky fluid.

A third group of tumours, totalling 60 and taken from 27 rats, consisted of fibro-adenomata (Fig. 4). These varied greatly in their structure, ranging from neoplasms with a large epithelial component to tumours which were almost pure fibromata and in which a considerable search had to be made to find any epithelial elements. In some of the fibro-adenomata it was possible to see that the epithelial component was either of non-secreting or of secreting type but it was not possible to subdivide the whole group of fibro-adenomata on this basis. Macroscopically, the fibro-adenomata were usually clearly distinguishable from the adenomata, being flabby instead of firm on palpation, and tough and stringy when cut.

In addition to these three main types of tumour two rats developed an anaplastic mammary tumour, composed of very primitive looking cells.

Many of the rats kept for relatively long periods developed tumours of more than one type, milk-secreting adenomata and fibro-adenomata frequently occurring together, and in an occasional animal all three types of tumour, non-secreting adenoma, secreting adenoma and fibro-adenoma, were found to have developed. Moreover, even within a single tumour the characteristics of more than one type of neoplasm were occasionally seen (Fig. 7).

The times when the tumours developed are shown in Fig. 9. It will be seen that in general the tumours which developed earliest, 8–16 weeks after the carcinogen-feeding, were the non-secreting adenomata, even though some adenomata of this nature did not appear until considerably later. The milk-secreting adenomata and the fibro-adenomata, apart from a few isolated exceptions, had a latent period of 6 months or more before they made their appearance.

The rate of growth varied considerably from tumour to tumour in each of the different types of neoplasm. Some tumours showed a rapid and continuous increase in size, attaining a diameter of up to 5 cm. Others grew more slowly, and some of these after a certain period of growth showed no further increase in size. A spontaneous decrease in the size of a tumour was not observed.

Two rats developed large tumours in the abdomen. These grew very quickly, and were found histologically to be of undifferentiated type. In one animal the tumour had apparently destroyed the right kidney and adjacent structures; in the other there were three large tumours in the mesentery. Only one of these rats had a palpable mammary tumour; this was of mixed type, being partly adenomatous and partly anaplastic. With this possible exception, distant metastases were not found.

FIG. 9.—An analysis of the mammary tumours which developed in 40 rats after a single dose of DMBA, with the times when they were first observed. The large symbols denote tumours observed on palpation; the small symbols indicate tumours too small to be palpable through the skin, but found to be present at necropsy. (For simplicity, in the few instances in which more than one type of neoplastic tissue was present in a single tumour, each component is recorded in the appropriate category as though it had been a separate tumour.)

*Response of tumours to hypophysectomy or ovariectomy*

Six rats with well established tumours were subjected to hypophysectomy. The nine tumours in these animals were all adenomata of the non-secreting type and after autopsy, 6–47 weeks after operation, all these tumours showed microscopic evidence of regression; in seven of the neoplasms the regression was complete, but in the other two only part of the tumour had regressed. The characteristic features of regression in these tumours were similar to those reported in our previous studies (Daniel and Prichard, 1963, 1964*a*). Two of these rats developed fibro-adenomata some weeks after hypophysectomy.

Ovariectomy was performed in 15 rats bearing macroscopically visible tumours. The latter included neoplasms of all the types mentioned above, but the only instances of regression found post-operatively in these animals occurred in non-secreting adenomata. Such tumours showed complete regression in 4 rats, partial regression in 3 others, and no regression in one animal. Regression did not occur in any of the numerous secreting adenomata or fibro-adenomata, although in a few of the latter the epithelial component appeared to have diminished after operation. Moreover, in many of the rats additional secreting adenomata and fibro-adenomata developed after the ovaries had been removed. There was one anaplastic mammary tumour present in this group of animals at the time of operation, and this did not regress.

### DISCUSSION

Our main object in giving carcinogen to rats has been to induce mammary tumours of a hormone-dependent type, so that we could compare the response of such neoplasms to various procedures which in one way or another modify their hormonal environment, such as hypophysectomy (Daniel and Prichard, 1963), transection of the pituitary stalk (Adams, Daniel and Prichard, 1963*a*, *b*, 1964 ; Cowie *et al.*, 1963, 1964 ; Daniel, Duchen and Prichard, 1964*a*, *b*, *c* ; Daniel and Prichard, 1963, 1964*b*), ovariectomy (Daniel and Prichard, 1964*a*) and adrenalectomy. This aim had been achieved in our previous studies in which 3-methylcholanthrene (3-MC) had been used as the carcinogen (Daniel and Prichard, 1961, 1964*c*). The tumours induced by 3-MC were almost exclusively of one type (non-secreting adenomata), and the finding that hypophysectomy caused regression in a high proportion of cases (Daniel and Prichard, 1963) indicated that most of the tumours were hormone-dependent. For our original purpose the present experiments, using DMBA, proved to be somewhat disappointing, because of the variety of tumours which developed. However, the fact that three different types of mammary tumours can be induced in large numbers by one carcinogen, and even in one animal, is of considerable theoretical interest, particularly since the development of the various tumours seems to be related to the time interval since administration of the carcinogen. (In our colony of Sprague-Dawley rats, many of which have been kept for over 2 years, the occurrence of spontaneous mammary tumours has been extremely rare.)

One of the three varieties of tumour induced by DMBA was what we have termed a non-secreting adenoma, to distinguish it from another type of adenoma, characterised by milk secretion. (We have used the term adenoma, rather than carcinoma or adenocarcinoma, terms used by others (Dao and Sunderland, 1959 ; Huggins *et al.*, 1959, 1961 ; Sterental *et al.*, 1963 ; Young, Cowan and Sutherland, 1963) for similar tumours because, although the intense mitotic activity often seen suggests malignancy (Foulds, 1961), the tumours do not give rise to distant metastases). These adenomata formed the smallest group of the three tumour types and were in general the earliest tumours to develop, most of them appearing within 6 months of the carcinogen feeding. Microscopically they were very similar in appearance to the adenomata induced by 3-MC, although the papillary pattern was more frequently seen after DMBA than after 3-MC. The degree of regression shown by these tumours after hypophysectomy or ovariectomy was very similar to that of adenomata induced by 3-MC (Daniel and Prichard, 1963, 1964*a*), and it would thus seem that the tumours of this type are essentially hormone-dependent.

However the great majority of the tumours induced by DMBA consisted of fibro-adenomata and milk-secreting adenomata. The fibro-adenomata, seen only occasionally in rats given 3-MC, formed the largest group of tumours in the present experiments, but the milk-secreting adenomata were almost as numerous, and this type of tumour we have not seen in any rat fed with 3-MC. Most of the tumours of these two types did not develop until 6 months or more after the DMBA had been given. In size these tumours ranged from very large neoplasms to plaques so small that they were only discovered in sections of what appeared macroscopically to be normal mammae. The histological features were the same in both the large and the small tumours.

The finding of very small tumours of all three types at autopsy was unexpected and stresses the need for a microscopic study of apparently normal mammary tissue in rats which have been fed with such a carcinogen. We did not examine all the mammary tissue in our rats, and thus the number of very small tumours recorded is almost certainly less than the number actually present. Whether these small neoplasms were newly-formed tumours or whether they had developed at an earlier stage and failed to grow to any size is not known. The rate of growth of the tumours which attained palpable size was variable and was not related to the type of neoplasm ; most of them showed continuous growth but some appeared to stop increasing in size after a time. We did not, however, observe a decrease in the size of any tumour occurring spontaneously, as reported by Young and Cowan (1963).

The data provided by the present series of experiments is not sufficient to show whether the fibro-adenomata and the milk-secreting adenomata are hormone-dependent or not. However, it seems probable that if there are hormonal influences associated with the growth of these tumours these are not the same as those which stimulate the growth of the non-secreting adenomata. For, whereas the growth of a non-secreting adenoma was usually inhibited by removal of the ovaries, in no instance did ovariectomy cause an already existing fibro-adenoma or milk-secreting adenoma to regress, nor did this operation prevent the subsequent development of new tumours of these types. By chance, the rats on which a hypophysectomy was performed did not include any animals bearing either a fibro-adenoma or a milk-secreting adenoma. Thus we have at present no data as to whether these tumours regress after removal of the pituitary. However, the possibility that the pituitary does have an influence on the development of at least some of these tumours is suggested by the finding that in the rats bearing non-secreting adenomata which had been subjected to hypophysectomy no milk-secreting adenomata, and only two fibro-adenomata, developed after the operation, even though some of these animals were kept for 28–44 weeks from the date of the carcinogen feeding. At a similar period of time milk-secreting adenomata and fibro-adenomata were developing in the rats with intact pituitaries. Moreover, as regards the milk-secreting adenomata, it seems likely on theoretical grounds that the development of these tumours is dependent on pituitary influence, since all the evidence at present available points to the fact that most of the hormones needed to induce lactation in the normal mammary gland are derived from cells in the anterior lobe of the pituitary gland (Cowie *et al.*, 1964). One of the characteristics of the milk-secreting adenoma was its general microscopic resemblance to a normal mammary gland during late pregnancy or lactation, and it therefore seems probable that in the rats which developed this type of tumour the carcinogen had caused a condition in which the pituitary was stimulated to secrete the hormones that are associated with lactation.

In view of the large number of milk-secreting adenomata that occurred in our rats given DMBA, it is surprising that this type of tumour is not mentioned by other workers who have used this carcinogen (Huggins and Yang, 1962; Sterental *et al.*, 1963; Young *et al.*, 1963). Indeed, the only account of milk-secreting tumours which we have been able to find is that of Foulds (1956), who observed this type of neoplasm occurring spontaneously in the mammary glands of a high-cancer strain of mice. In his animals, however, the tumours developed only during pregnancy or lactation, and thus the conditions were quite different from those in our rats, none of which was given the opportunity to become pregnant.

There are few reports in the literature of mammary fibro-adenomata developing after the oral administration of carcinogenic hydrocarbons. This type of tumour was one of the less common varieties of neoplasm induced by methylcholanthrene in the experiments of Shay, Harris and Gruenstein (1952), and these workers suggested that the development of a fibro-adenoma, instead of a purely glandular tumour, was the result of an approximate balance having become established between the amounts of oestrogen and testosterone present in the animal. Fibro-adenomata occurred only occasionally in our experiments with 3-MC, and are not reported by Huggins *et al.* (1959) or by Dao and Sunderland (1959) as having developed in their rats given this carcinogen. It would thus seem that only a very few of the tumours induced by 3-MC are fibro-adenomata. The present experiments indicate that this is not the case when the carcinogen used is DMBA, and Huggins and Yang (1962) also report that fibro-adenomata developed in many of their rats given DMBA. On the other hand, some workers' accounts of the tumours induced by this carcinogen contain no mention of fibro-adenomata (Sterental *et al.*, 1963; Young *et al.*, 1963).

The exact mechanism by which tumours are induced by oral administration of carcinogenic hydrocarbons is not known, but it is clear that the carcinogen is not the sole factor in the causation of the tumours. The conditions in the host, including hormonal factors, must play an important role, and it seems possible that the hormonal conditions prevailing in an individual animal at the time when the carcinogen is exerting its effect determine the type of tumour which develops. In this connection it may be significant that in the present experiments most of the non-secreting adenomata developed within 6 months of the carcinogen feeding, i.e. while the rats were relatively young, whereas the great majority of the milk-secreting adenomata and fibro-adenomata did not appear until from 6 to 12 months had elapsed since the carcinogen had been given, i.e. in older animals.

### SUMMARY

Mammary tumours were induced in female Sprague-Dawley rats by a single feeding of 20 mg. 9,10-dimethyl-1,2-benzanthracene, given at the age of 50 days. The tumours were of three main types: ordinary adenomata resembling those induced by 3-methylcholanthrene, adenomata of an unusual type characterised by milk-secretion, and fibro-adenomata. The ordinary, non-secreting adenomata formed a minority of the tumours, and usually developed within 6 months of the carcinogen feeding. The milk-secreting adenomata and the fibro-adenomata were the tumours which developed in largest numbers, but most of the tumours of this type did not develop until 6–12 months after the carcinogen had been given. The growth of the non-secreting adenomata was influenced by the pituitary and to some extent by the ovaries.

This work was supported by a grant from the British Empire Cancer Campaign for Research for which we are most grateful. We wish to thank Mr. E. Bernard, F.A.T.A., and Miss J. Booty for their help with the care of the animals; Miss E. Sporle for photographic assistance and Mrs. J. Storms and Miss C. Haseler for cutting the sections of the tumours and other tissues.

### REFERENCES

ADAMS, J. H., DANIEL, P. M. AND PRICHARD, M. M. L.—(1963a) *Quart. J. exp. Physiol.*, **48**, 217.—(1963b) *Acta endocr., Copenhagen*, **43**, Supplement **81**.—(1964) *J. Path. Bact.*, **87**, 1.

COWIE, A. T., DANIEL, P. M., KNAGGS, G. S., PRICHARD, M. M. L. AND TINDAL, J. S. —(1964) *J. Endocrin.*, **28**, 253.

*Idem*, DANIEL, P. M., PRICHARD, M. M. L. AND TINDAL, J. S.—(1963) *Ibid.*, **28**, 93.

DANIEL, P. M., DUCHEN, L. W. AND PRICHARD, M. M. L.—(1964a) *J. Path. Bact.*, **87**, 385.—(1964b) *Quart. J. exp. Physiol.*, **49**, 235.—(1964c) *Ibid.*, **49**, 243.

*Idem* AND PRICHARD, M. M. L.—(1961) *Brit. J. Cancer*, **15**, 828.—(1963) *Ibid.*, **17**, 446.—(1964a) *Ibid.* **17**, 687.—(1964b) *Acta endocr., Copenhagen*, **45**, 84.—(1964c), *Nature, Lond.*, **201**, 578.

DAO, T. L. AND SUNDERLAND, H.—(1959) *J. nat. Cancer Inst.*, **23**, 567.

FOULDS, L.—(1956) *Ibid.*, **17**, 783.—(1961) 'The Development of Tumors'. *In* Proceedings of the Fourth National Cancer Conference, pp. 85–89, J. B. Lippincott Co.

HUGGINS, C., BRIZIARELLI, G. AND SUTTON, H.—(1959) *J. exp. Med.*, **109**, 25.

*Idem*, GRAND, L. C. AND BRILLANTES, F. P.—(1961) *Nature, Lond.*, **189**, 204.

*Idem* AND YANG, N. C.—(1962) *Science*, **137**, 257.

SHAY, H., HARRIS, C. AND GRUENSTEIN, M.—(1952) *J. nat. Cancer Inst.*, **13**, 307.

STERENTAL, A., DOMINGUEZ, J. M., WEISSMAN, C. AND PEARSON, O. H.—(1963) *Cancer Res.*, **23**, 481.

YOUNG, S. AND COWAN, D. M.—(1963) *Brit. J. Cancer.*, **17**, 85.

*Iidem* AND SUTHERLAND, L. E.—(1963) *J. Path. Bact.*, **85**, 331.


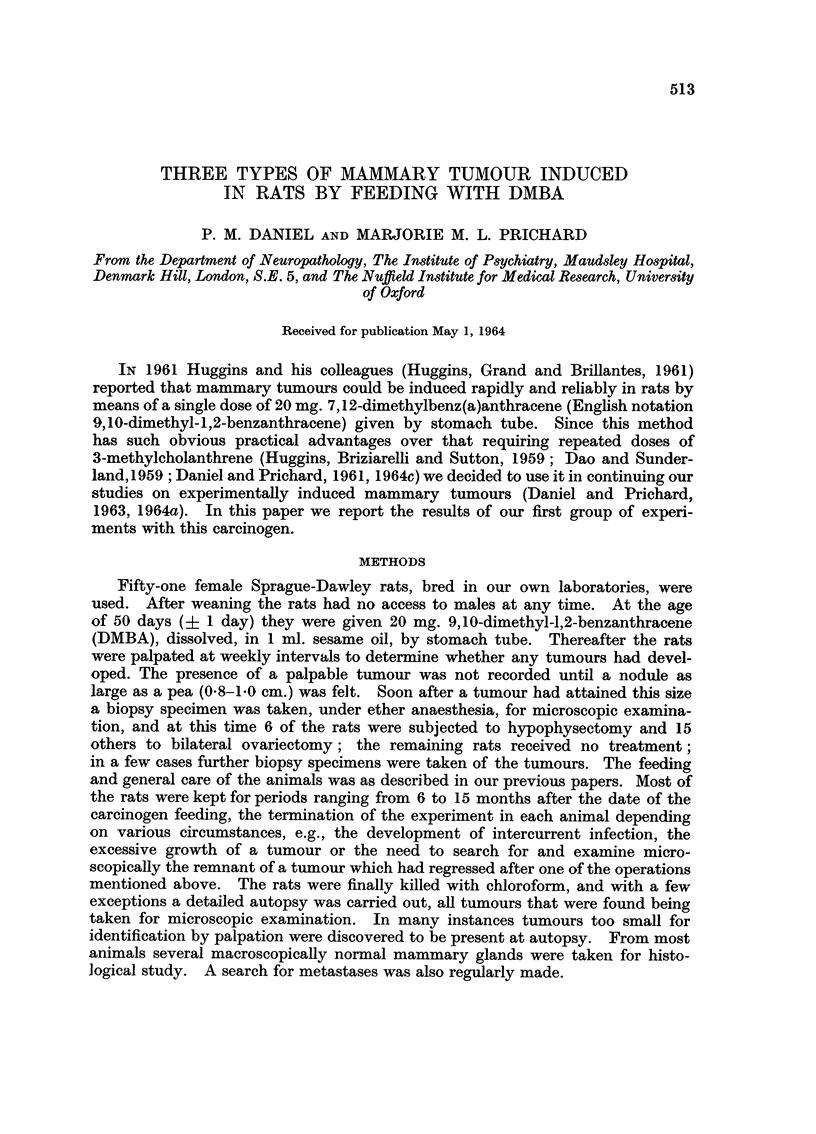

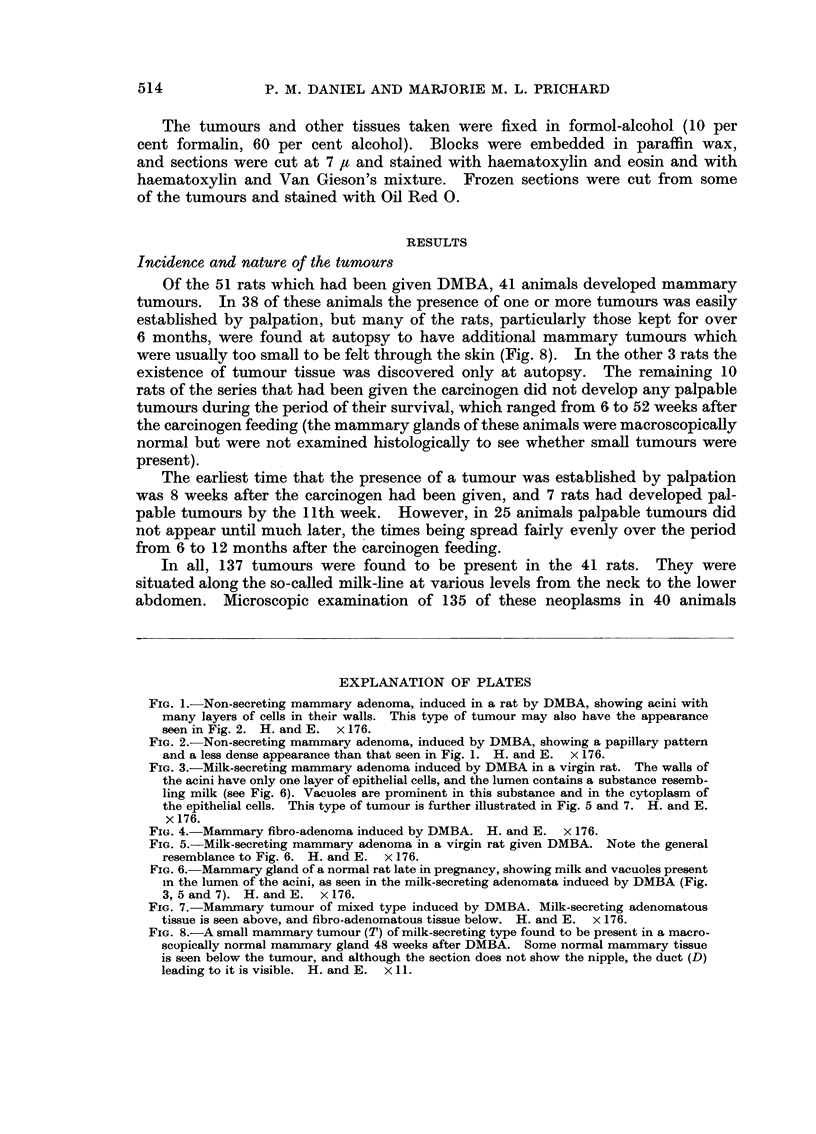

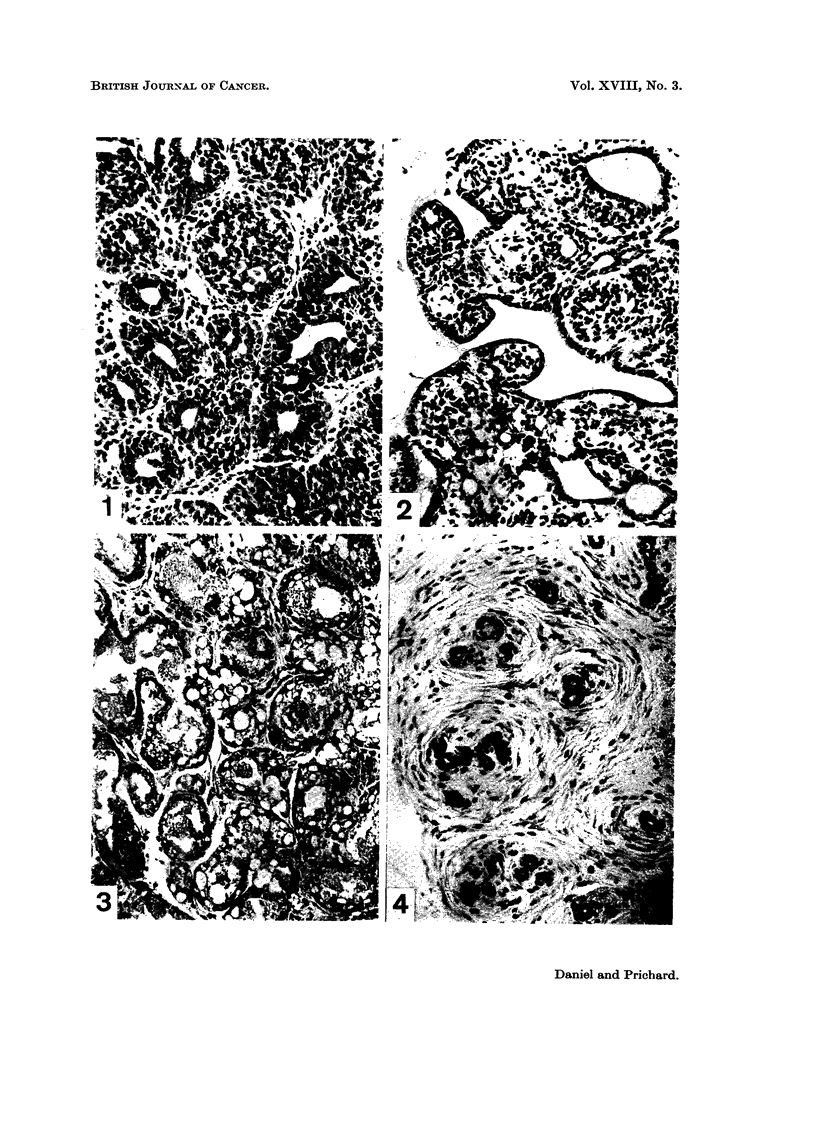

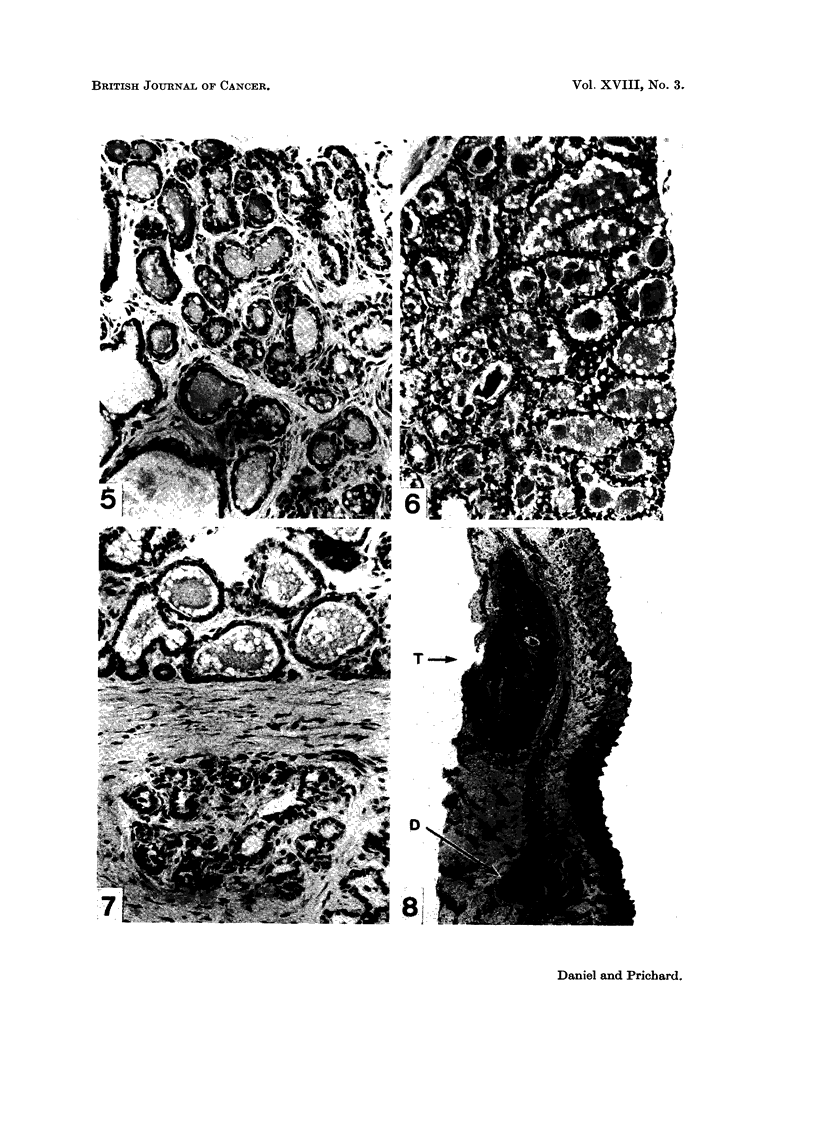

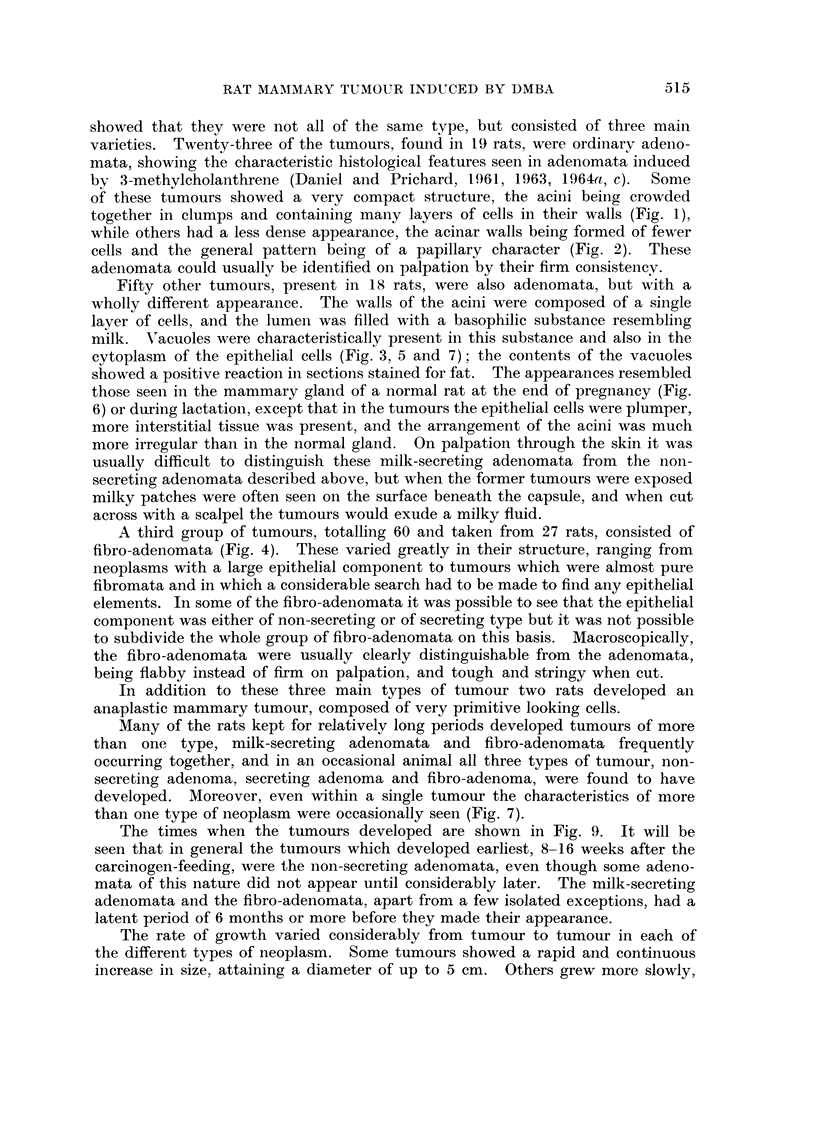

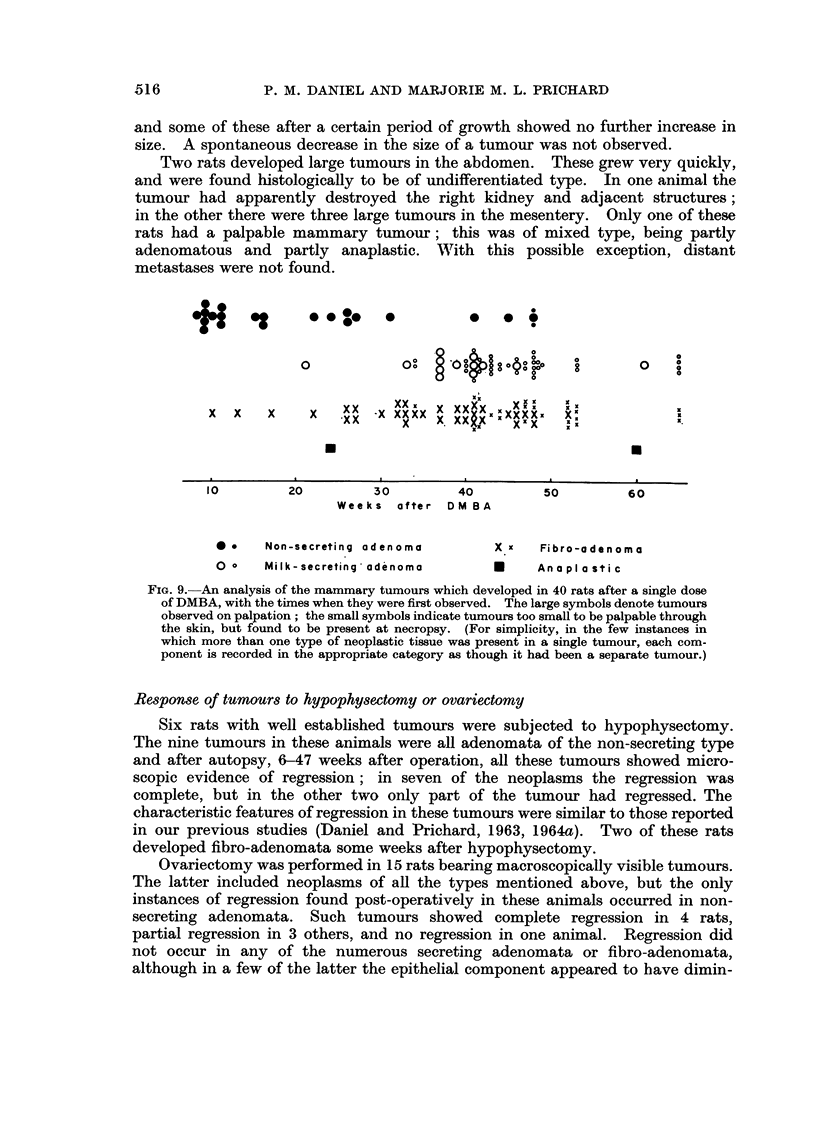

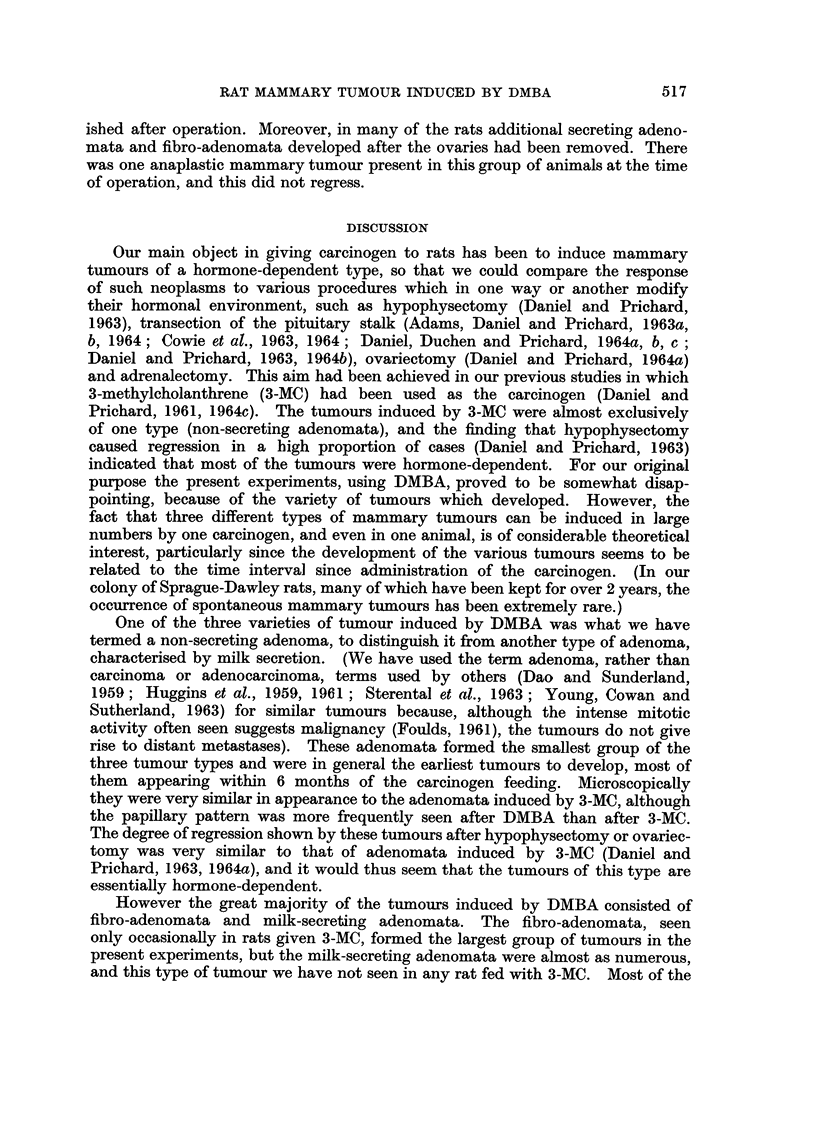

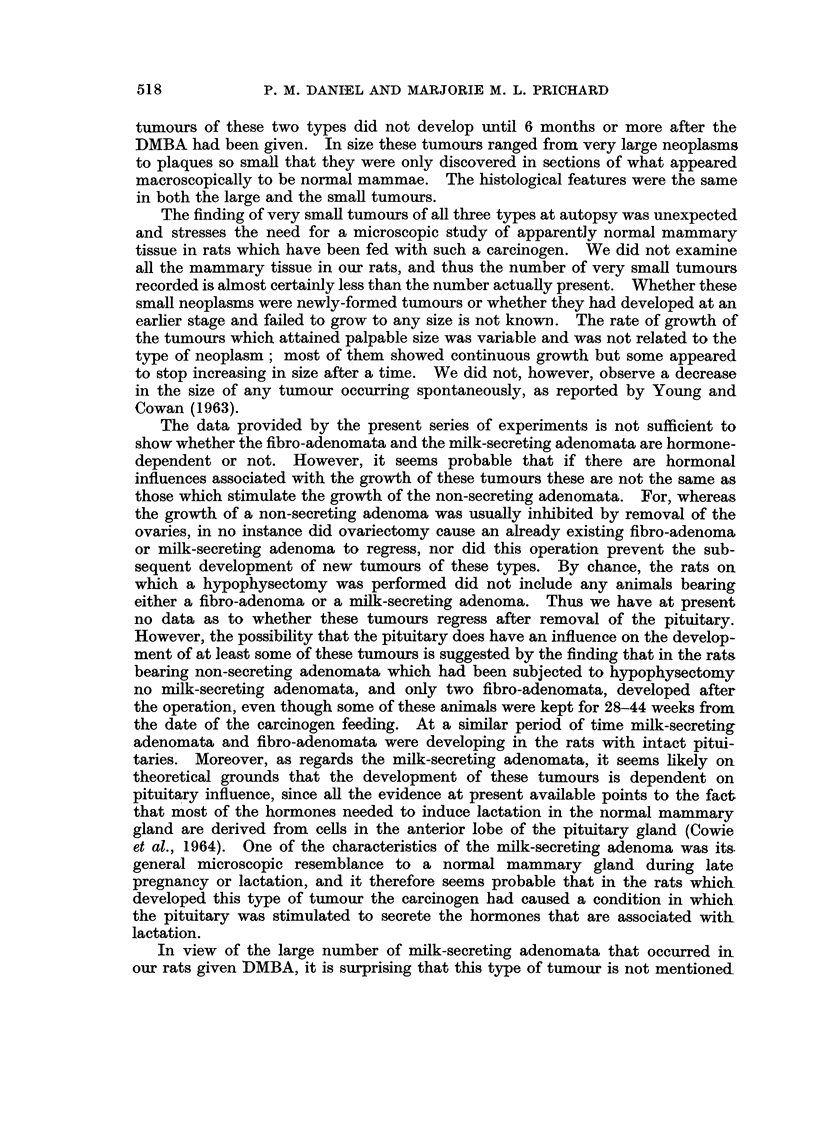

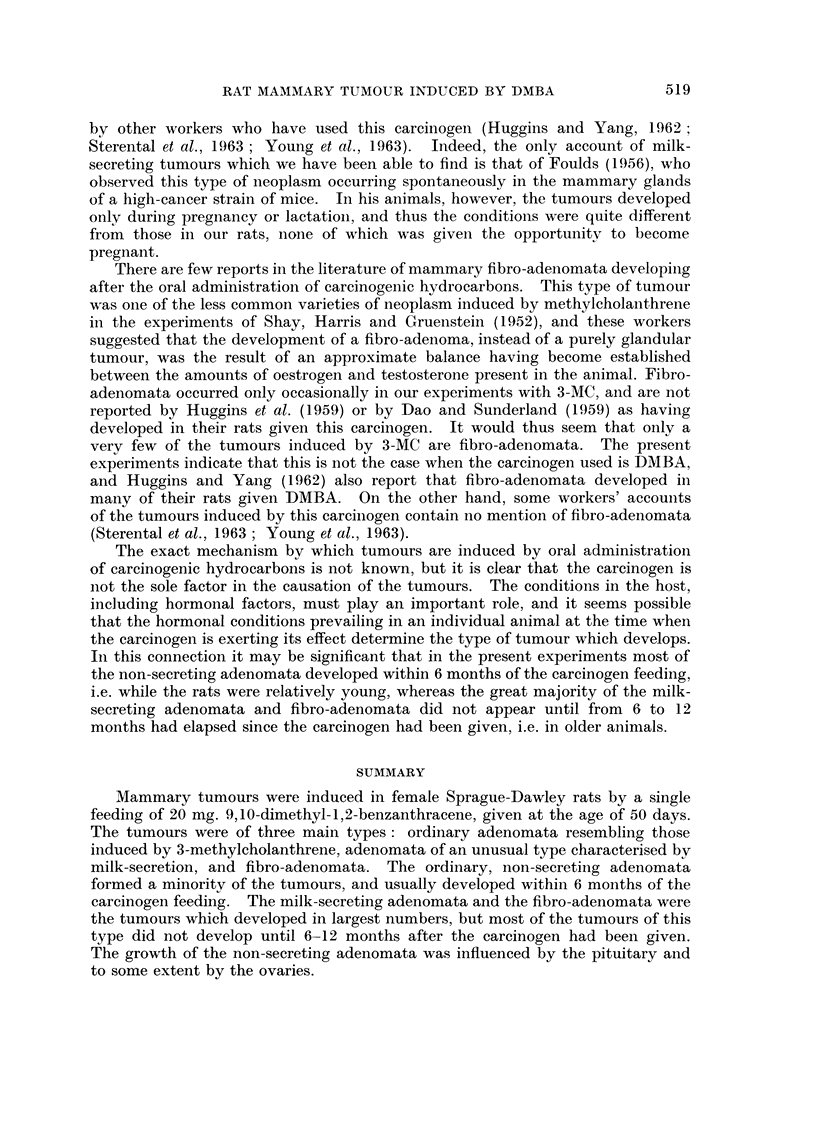

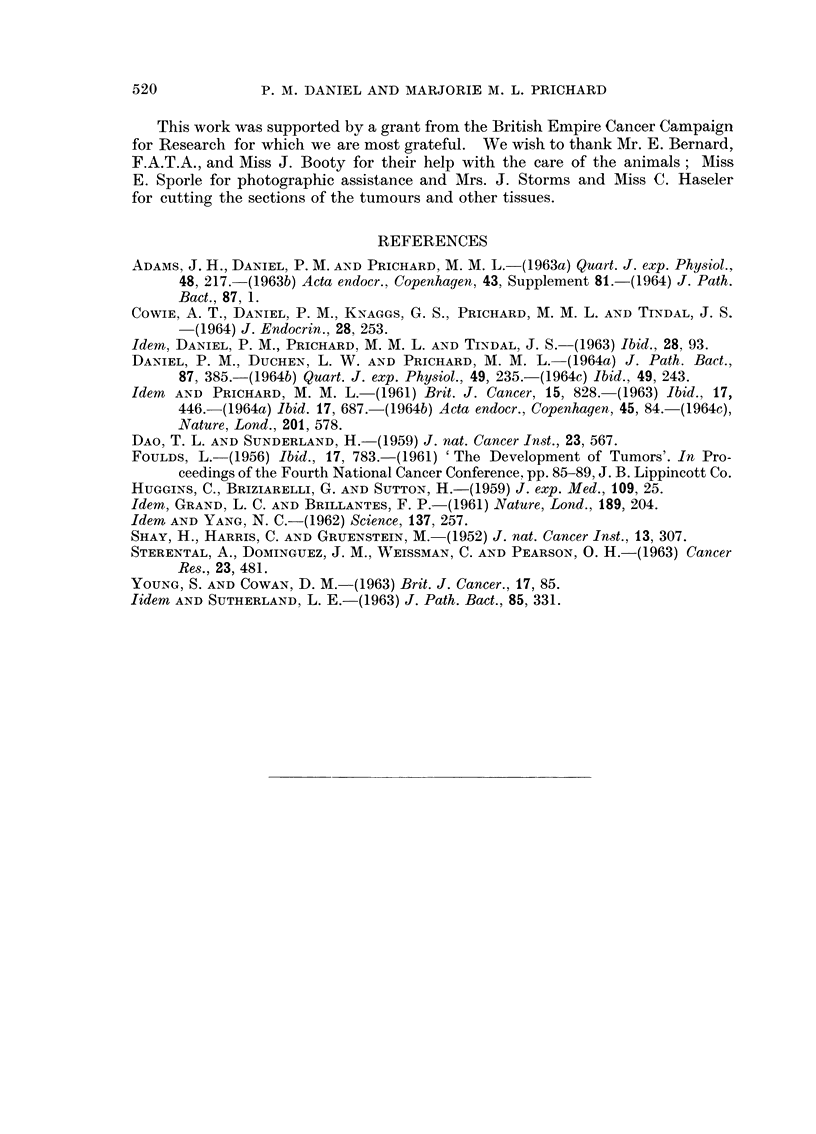


## References

[OCR_00448] ADAMS J. H., DANIEL P. M., PRICHARD M. M. (1963). The volumes of pars distalis, pars intermedia and infundibular process of the pituitary gland of the rat, with special reference to the effect of stalk section.. Q J Exp Physiol Cogn Med Sci.

[OCR_00453] COWIE A. T., DANIEL P. M., KNAGGS G. S., PRICHARD M. M., TINDAL J. S. (1964). LACTATION IN THE GOAT AFTER SECTION OF THE PITUITARY STALK.. J Endocrinol.

[OCR_00459] DANIEL P. M., DUCHEN L. W., PRICHARD M. M. (1964). THE CYTOLOGY OF THE PITUITARY GLAND OF THE RHESUS MONKEY: CHANGES IN THE GLAND AND ITS TARGET ORGANS AFTER SECTION OF THE PITUITARY STALK.. J Pathol Bacteriol.

[OCR_00466] DAO T. L., SUNDERLAND H. (1959). Mammary carcinogenesis by 3-methylcholanthrene. I. Hormonal aspects in tumor induction and growth.. J Natl Cancer Inst.

[OCR_00472] HUGGINS C., GRAND L. C., BRILLANTES F. P. (1961). Mammary cancer induced by a single feeding of polymucular hydrocarbons, and its suppression.. Nature.

[OCR_00473] HUGGINS C., YANG N. C. (1962). Induction and extinction of mammary cancer. A striking effect of hydrocarbons permits analysis of mechanisms of causes and cure of breast cancer.. Science.

[OCR_00475] SHAY H., HARRIS C., GRUENSTEIN M. (1952). Influence of sex hormones on the incidence and form of tumors produced in male or female rats by gastric instillation of methylcholanthrene.. J Natl Cancer Inst.

[OCR_00477] STERENTAL A., DOMINGUEZ J. M., WEISSMAN C., PEARSON O. H. (1963). Pituitary role in the estrogen dependency of experimental mammary cancer.. Cancer Res.

[OCR_00481] YOUNG S., COWAN D. M., SUTHERLAND L. E. (1963). The histology of induced mammary tumours in rats.. J Pathol Bacteriol.

